# Intrauterine growth patterns in rural Ethiopia compared with WHO and INTERGROWTH-21^st^ growth standards: A community-based longitudinal study

**DOI:** 10.1371/journal.pone.0226881

**Published:** 2019-12-31

**Authors:** Meselech Roro, Wakgari Deressa, Bernt Lindtjørn

**Affiliations:** 1 Centre for International Health, University of Bergen, Bergen, Norway; 2 Department of Reproductive Health and Health Service Management, School of Public Health, College of Health Sciences, Addis Ababa University, Addis Ababa, Ethiopia; 3 Department of Preventive Medicine, School of Public Health, College of Health Sciences, Addis Ababa University, Addis Ababa, Ethiopia; London School of Hygiene and Tropical Medicine, UNITED KINGDOM

## Abstract

**Introduction:**

Children’s well-being is highly influenced by their fetal growth. Adequate intrauterine growth (IUG) is a basic feature of a healthy pregnancy. The aim of our study was to assess IUG patterns in a rural and drought-affected population in the Rift Valley area of the Adami Tullu district in Oromia, Ethiopia.

**Methods:**

We conducted a longitudinal, community-based study of IUG patterns utilizing serial ultrasound measurements. Data were collected for 17 months, from July 2016 to November 2017. We included 675 singleton foetuses ≤ 24 weeks old, based on ultrasound-derived estimates of gestational age, and followed them until delivery. We obtained head circumference, biparietal diameter, abdominal circumference, femur length, and estimated fetal weight at 26, 30, and 36 weeks. Fetal weight was estimated using the Hadlock algorithm, and the 5^th^, 10^th^, 25^th^, 50th, 75^th^, 90^th^, and 95^th^ centiles were developed from this model. We compared the biometric measurements and fetal weight data from our study to the World Health Organization (WHO) and INTERGROWTH-21^st^ fetal growth reference standards.

**Results:**

Distribution of the biometric measurements and estimated fetal weights in our study were similar to those for the WHO and INTERGROWTH-21^st^ references. Most measurements were between -2 and +2 of the reference z-scores. Based on the smoothed percentiles, the 5^th^, 50^th^, and 95^th^ percentiles of our study had similar distribution patterns to the WHO chart, and the 50^th^ percentile had a similar pattern to the INTERGROWTH-21^st^ chart.

**Conclusions:**

Our study determined fetal growth patterns in a drought-affected rural community of Ethiopia using common ultrasound biometric measurements. We found similar IUG patterns to those indicated in the WHO and INTERGROWTH-21^st^ fetal growth reference standards.

## Introduction

Children’s well-being is highly influenced by fetal growth. Adequate intrauterine growth (IUG) is a basic feature of a healthy pregnancy. Abnormal fetal growth has been associated with adulthood diseases, such as stunting, cardiovascular disease, diabetes mellitus, and obesity [1, 2]. Ultrasound imaging constitutes an indispensable tool for estimating gestational age and fetal size. Fetal growth assessment using ultrasound improves detection of IUG restrictions and helps identify those foetuses at risk of sickness during the neonatal period [3]. Since the early 1980s, several ultrasound-based fetal weight references have been published [4]. Most are based on cross-sectional or retrospective studies [3, 5, 6], with only one observation from each woman. Moreover, these references include fetal size only and thus provide limited information about fetal growth. Prospective studies, in comparison, can confirm gestational age and assess fetal growth via repeated measurements [7]. Some earlier longitudinal ultrasound studies were conducted using small sample sizes, and those with larger sample sizes were performed on predominantly Caucasian populations [8]. Evidence from some of these studies shows that differences in fetal growth depend on racial category, sex, parity, and maternal size [8].

Two recent multicentre studies that have been used to develop IUG standards are the World Health Organization (WHO) Multicentre Growth Reference Study and the INTERGROWTH-21^st^ project [9, 10]. Other “birth weight-for-gestational-age references” have been developed with large, mostly population-based databases, and they provide birth weight percentiles by gestational week [3, 11]. The WHO study, which identifies differences in estimated fetal weight (EFW) between countries, finds that maternal age, parity, height, and weight only partially explain the differences [9]. Both the WHO and INTERGROWTH-21^st^ studies measure biometric growth, although INTERGROWTH-21^st^ does not present percentile data for EFW. The INTERGROWTH-21^st^ study estimates fetal weight during ultrasound using head circumference and abdominal circumference, whereas the WHO study uses the Hadlock 3 algorithm to generate EFW using head circumference, abdominal circumference, and femoral length. Both standards can be used as references for interpreting routine ultrasound measurements.

Because these studies were conducted on presumably healthy and well-nourished pregnant women, the risk of adverse perinatal outcomes in areas such as Ethiopia, which is repeatedly affected by drought and food insecurity, remains unclear. Poor maternal nutrition could be an important contributor to IUG restriction and subsequent low birth weight [12, 13]. Furthermore, the WHO study recommends the establishment of population-specific fetal growth charts [9]. As Intrauterine growth restriction (IUGR) is reportedly high in African countries [14], a fetal growth chart that reflects reliable data from the local population needs to be developed. This chart will serve as a basis to verify if the current growth charts can be used to assess populations in Africa. We hypothesised that the IUG pattern in our study site would differ from those of the WHO and INTERGROWTH-21^st^ Project. The aim of our study thus was to assess IUG patterns in a rural and drought-affected population in Ethiopia.

## Materials and methods

### Study design and settings

This longitudinal, ultrasound-based study was conducted in the rural Adami Tullu district in the East Shewa Zone of the Oromia Regional State in Ethiopia. The district is in the Rift Valley area of south-central Ethiopia. According to the 2007 National Census, the projected population of Adami Tullu was approximately 177,390 people in 2015 [15]. We followed women residing in the district within the context of a recently conducted large, community-based, cluster-randomized, controlled trial to determine how the combined use of long-lasting insecticidal nets and indoor residual spraying affected malaria incidence compared with nets alone or spraying alone in the rural part of the district [16].

### Study population

We included pregnant women living in the study district’s villages or in the clusters of the previously mentioned malaria trials and followed them until delivery. Pregnant women were identified by data collectors, who went house to house interviewing every woman of reproductive age (15–49 years) to ask if she was pregnant. If a woman responded that she was not pregnant or did not know her pregnancy status, the data collectors used a WHO checklist to determine with reasonable certainty that a woman was not pregnant [17]. For those women who were suspected to be pregnant based on their interview responses or the WHO checklist, an ultrasound scan was performed to verify the pregnancy and estimate gestational age. We then included all pregnant women with a gestational age of less than 24 weeks who agreed to participate in the study.

The sample size was determined based on birth weight, the primary outcome measure. The size should be adequate to detect a birth weight difference of 110 g, based on WHO and INTERGROWTH-21^st^ findings and on previous estimates of standard deviations of birth weight of approximately 500 g [9, 18]. Thus, the estimated sample size was 325 in each group (power of 80%). Allowing for a 5% non-response rate and 5% loss to follow-up, the total sample size was 716.

Enrolment was conducted from July 2016 to June 2017, and follow-up was completed in November 2017 when the last woman gave birth. All consenting pregnant women with a gestational age ≤ 24 weeks and who lived in the study area were included in the study. Those pregnant women who left the study area or who had histories of multiple pregnancy, abortion, congenital abnormalities, or intrauterine fetal death were excluded from the analysis.

### Data collection

After enrolment, we collected data on baseline socioeconomic status, maternal age, obstetric history, occurrence of chronic diseases, anthropometric measurements (e.g. maternal weight, height, and mid-upper-arm circumference), blood pressure, haemoglobin, and rapid diagnostic test results for malaria. Conditions that could affect fetal growth and birth weight, such as twin pregnancy, congenital malformation, hypertensive disorders, malnutrition, anaemia, and malaria were assessed. Testing for maternal diabetes, HIV, and syphilis was not performed, but these data were collected from the history reported by those women who received antenatal care at health facilities. All assessments were conducted at nearby health posts. The women were then given a follow-up card to visit the health posts to attend three prescheduled visits at 26, 30, and 36 weeks of gestation. Data collectors visited the women’s homes one day prior to their appointments to remind them.

During each scheduled visit, we recorded the ultrasound results, anthropometric measurements, blood pressure, haemoglobin measurements, and malaria test results. Haemoglobin was measured using a portable spectrophotometer (HemoCue, Ängelholm, Sweden). Anaemia was defined as a haemoglobin concentration of less than 11 g/dl [19]. Malaria infection was tested using a rapid diagnostic test (Parascreen TM Zephyr Biomedicals, Goa, India; Paracheck Orchid Biomedical Systems, Goa, India; or ParaHIT Span Diagnostics Ltd., Surat, India). Blood pressure was measured using a digital measuring machine (BOSCH+ SOSH, Germany). A mother was considered to be hypertensive when systolic blood pressure exceeded 140 mmHg or diastolic pressure exceeded 90 mmHg [20]. Maternal weight was determined using a digital weighing scale (Coline) to the nearest 100 gm. We measured the height of each pregnant woman at baseline using a standard wooden board. The mid-upper-arm circumference (MUAC) was determined using a standard MUAC tape. Circumferences of less than 23 cm indicated maternal malnutrition [21]. According to the Ethiopian Targeted Supplementary Feeding Programme guideline, pregnant mothers with an MUAC below 23 cm should receive food supplements to treat moderate malnourishment [22].

Ultrasound examination was performed using portable SonoSite M-Turbo diagnostic imaging and a full-colour, flow-mapping ultrasound system (FUJIFILM SonoSite Inc.,Bothell, WA 98021 USA) at enrolment and each subsequent visit. We used ultrasound to estimate gestational age for all pregnancies at enrolment and at each scheduled visit [23]. At enrolment, gestational age and estimated date of delivery were calculated utilizing the formula from Hadlock et al., which uses biparietal diameter (BPD), head circumference (HC), abdominal circumference (AC), and femur length (FL) [24]. Crown rump length was used to estimate gestational age until 13 weeks and 4 days of pregnancy (fetal length ≤ 75 mm). The average of two measurements was calculated for each biometric parameter and recorded at enrolment and at the three scheduled visits. Fetal weight was estimated using the Hadlock algorithm for BPD, HC, AC, and FL [25]. Biometric measurements were assessed based on the standards [26]. Birth weights were measured using a digital infant weighing scale (Beurer Digital Baby Scale). Newborn birth weight, sex, head and chest circumference, and length were recorded by trained nurses within 72 hours of delivery, whether at home or at a facility. The nurses visited the pregnant mothers regularly until they gave birth.

### Data quality assurance

Thirteen trained nurses conducted the anthropometric measurements and blood tests for anaemia and malaria. We provided these nurses with two days of training on anthropometry measurement techniques prior to the start of the study. Intra- and inter-technical measurement errors were checked, and repeated training was given until the measurements reached the recommended cut-off points (largest acceptable differences between repeated measurements of 1.0 cm for height and 0.5 cm for MUAC) [27]. The intra-technical error was 0.38 cm for height and 0.32 cm for MUAC, and the inter-technical error was 0.37 cm for height and 0.33 cm for MUAC. Ultrasound assessment was done by the first author, who was trained by a senior obstetrician and gynaecologist at St. Paul Hospital Millennium Medical College. The author’s training was validated at Tikur Anbessa Specialized Teaching Hospital of Addis Ababa University. Based on biometric measurements of 27 foetuses, the calculated Pearson’s correlations (r) between the two independent measurements on the same foetus were r = 0.98 for BPD and HC, 0.96 for AC, and 0.89 for FL.

### Statistical analysis

Data were entered and cleaned using SPSS version 24 (SPSS Inc., Chicago, IL, USA) and analysed using Stata software version 15 (Stata Corp., College Station, TX, USA). A descriptive analysis was performed. Reference curves were estimated based on centiles for individual BPD, HC, AC, FL biometric parameters. EFW was derived at each gestational age from 24 to 38 weeks. The reference curve was fitted using centiles for reference models and the 5^th^, 10^th^, 25^th^, 50^th^, 75^th^, 90^th^, and 95^th^ centiles. The proportion of foetuses in the cohort that were small for gestational age was evaluated for weights that fell below the 10^th^ percentile for specific gestational age and the overall study population [13]. Fractions of estimated biometric measurements were computed to the nearest whole number for all constructed tables of EFW, BPD, HC, AC, and FL. The z-scores and percentiles of biometric measurements and EFW were calculated and fitted to INTERGROWTH-21^st^ International Standards for Fetal Growth [10]. Newborns weighing less than 2,500 g were categorized low birth weight [28]. Babies born alive before 37 weeks of pregnancy were considered preterm [29]. A mother was defined as malnourished if her MUAC fell below 23 cm. A household wealth index also was constructed using principal component analysis [30]. Fourteen household assets were used to calculate wealth: presence of electricity; ownership of a radio, mobile telephone, chair, table, bed, bicycle, or land; separate kitchen from living area; ownership of animals or animal carts; types of roof and walls; and presence of potable drinking water.

### Ethical considerations

The study received ethical approval from the institutional review board of the College of Health Sciences, Addis Ababa University, in June 2015 (ref.;005/15/SPH) and from the Regional Committee for Medical and Health Research Ethics, Western Norway (ref: 2013/986/REK Vest). Written permission to perform this study was obtained from the Oromia Regional Health Bureau and relevant provincial and local authorities in the district. Informed written consent was obtained from each participant, and all procedures were conducted based on voluntary participation. Pregnant women who had indication(s) for treatment were referred to a nearby hospital.

### Role of funding source

The funding sources for this study had no role in the design, data collection, analysis, interpretation of the results, or writing of the report.

## Results

**Fig 1:**
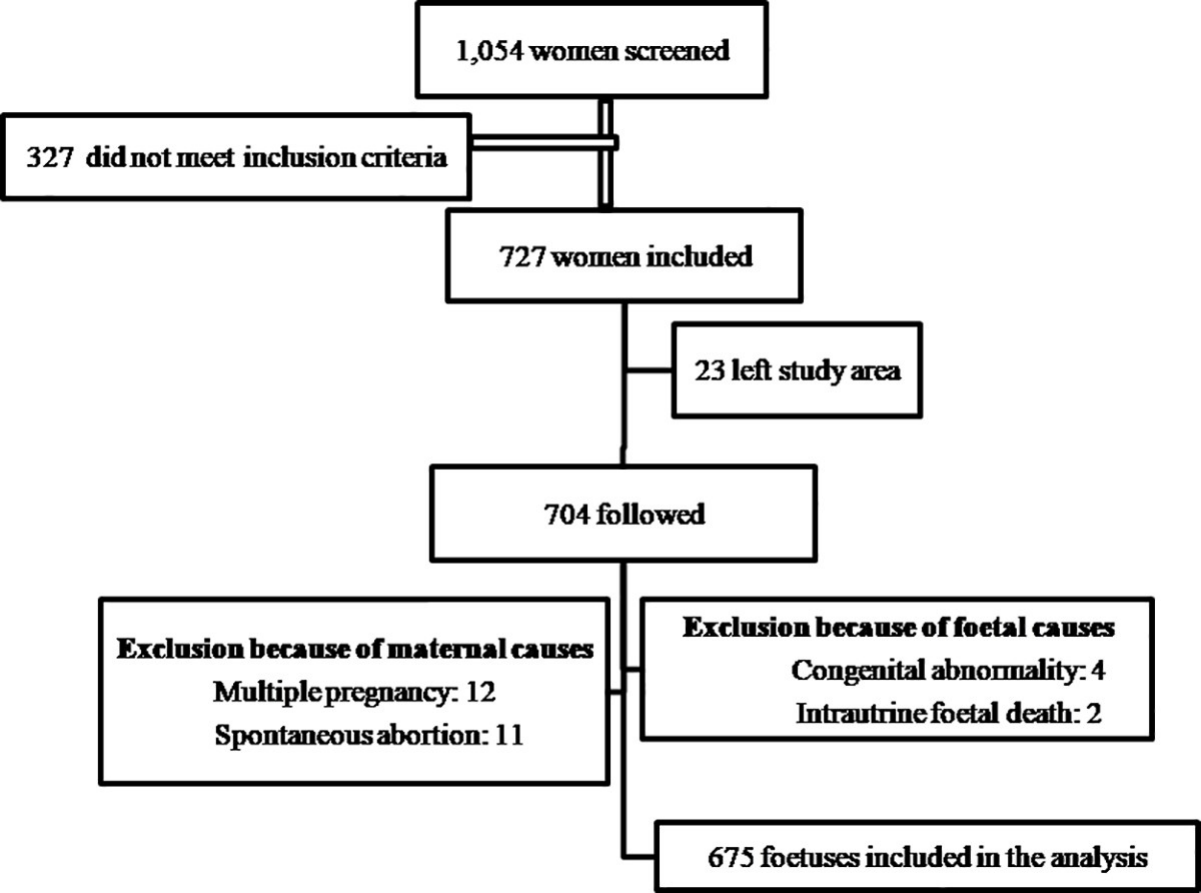
Flowchart of pregnant women included in the study and final analysis.

Out of 1,054 self-reported pregnant women, 727 met the inclusion criteria, as shown in Fig 1. Among those who did not meet inclusion criteria, 11 (3.0%) were not pregnant on ultrasound examination, and 316 (30%) exceeded 24 weeks of gestation. Among those who met the inclusion criteria, 23 (3.2%) left the study district; 15 (2.1%) had terminations, either spontaneously or due to medical indications related to congenital abnormalities and pregnancy complications; 12 (1.7%) were twins; and two experienced intrauterine fetal deaths. As shown in the S1 Table, we found no significant difference in the baseline characteristics of those who were lost to follow-up and those who were included in the final analysis. The final analysis comprised 1,808 ultrasound scans from 675 singleton foetuses. Growth patterns were analysed, and a growth chart was developed based on the EFW of these 675 singleton foetuses.

### Baseline characteristics

Table 1 shows the socio-demographic and economic characteristics of the study participants. The mean ± standard deviation (SD) of maternal age at enrolment was 24.9 ± 5.4 (15–45) years, the highest proportion of pregnancies occurring in the 15–24 age group. Family size of the study participants ranged from 2 to 14 (mean: 5.6) persons. All study participants were married, and 52% (351/675) did not have formal education. At baseline, the mean maternal height was 157.1 cm (133 cm to 178 cm) and mean body mass index (BMI) was 21.5 (range 14.8 to 31.5), with 10.9% (73/668) having a BMI under 18.5 kg/m^2^. At baseline, 40.6% (281) of the women had an MUAC under 23 cm, and 17.7% (114) of the women were anaemic (Table 1).

**Table 1:** Socio-demographic and economic characteristics of mothers who participated in the study (Adami Tullu district, Oromia, south-central Ethiopia, 2016–2017).

Variable		n (%)
Age in years (n = 673)	15–24	324 (48.0)
	25–34	306 (45.3)
	35–45	45 (6.7)
Education status (n = 671)	No formal education	351 (52.2)
	Primary school	264 (39.3)
	Secondary school	57 (8.5)
Ethnicity (n = 675)	Oromo	634 (94.4)
	Gurage	9 (1.3)
	Amhara	3 (0.5)
	Other	26 (3.9)
Religion (n = 675)	Muslim	582 (86.6)
	Orthodox	78 (11.6)
	Protestant	11 (1.6)
	Catholic	1 (0.2)
Occupation (n = 673)	Housewife	515 (76.3)
	Farmer	119 (17.6)
	House maid	15 (2.2)
	Other*	24 (3.9)
Wealth status (n = 675)	Poor	220 (32.8)
	Middle	228 (34.0)
	Rich	222 (33.1)
Family size (n = 675)	2–5	333 (49.3)
	>5	342 (50.7)
Haemoglobin (n = 665)	<110 g/dl	114 (17.7)
	<110 g/dl	551 (81.3)
Mean maternal height (cm) ± SD		157.1± 6.5
Mean maternal weight (kg) at baseline ± SD		53.1 ±6.9
Body mass index (n = 668)	Underweight	73 (10.9)
	Normal weight	533 (79.8)
	Overweight	62 (9.3)
Mean maternal MUAC (cm) at baseline ± SD		23.6 ±2.3

* Day labourer, tradesperson, fisher, student, government employee, and non-governmental organization employee

Most (84.9%; 573/675) study participants were multigravida, and 19.9% (134/675) had a history of abortion. At the start of the study, 87% (587/675) had a gestational age of 12 weeks or more. The majority (54%; 329/607) had their first antenatal care visit after 26 weeks of gestation: 29.8% (315) had two visits, and 33.5% (354) had three visits. A small number (0.7%; 5/675) of the mothers had malaria infection (3 had *plasmodium vivax*, 1 had *plasmodium falciparum*, and 1 was mixed species). Only one mother was diagnosed with hypertension. None reported to have diabetes, HIV, or syphilis (Table 2).

**Table 2:** Past and present obstetric and clinical characteristics of participating women and their newborns (Adami Tullu district, south-central Ethiopia, 2016–2017).

Variable*		n (%)
Gravida (n = 675)	Primigravida	102 (15.1)
	Multigravida	573 (84.9)
Parity (n = 675)	Nulliparous	121 (17.9)
	Multiparous	449 (66.5)
	Grand multiparous	105 (15.6)
Previous abortion history (n = 673)	Yes	134 (19.9)
	No	539 (80.1)
Gestational age at inclusion (n = 675)	< = 12 weeks	88 (13.0)
	>12–24 weeks	587 (87.0)
Place of delivery (n = 626)	Home	333 (53.1)
	Health post	2 (0.3)
	Health centre	148 (23.6)
	Hospital	143 (22.8)
Mode of delivery (n = 618)	Spontaneous vaginal delivery	607 (98.1)
	Assisted (forceps/vacuum) delivery	4 (0.6)
	Emergency Caesarean section	8 (1.3)
Sex of newborn (n = 623)	Male	363 (58.3)
	Female	260 (41.7)
Mean birth weight (g) (n = 610)		3214 (1292–5000)
Time of birth weight measurement (n = 610)	Within 24 hours after delivery	538 (88.2)
	Within 48 hours after delivery	68 (11.1)
	Within 72 hours after delivery	4 (0.7)
Birth status	Low birth weight (<2500 g) (%)	48/610 (7.9)
	Preterm birth (<37 weeks)	31/630 (4.9)
Malaria (n = 5)	*Plasmodium vivax*	3 (0.4)
	*Plasmodium falciparum*	1 (0.1
	Mixed species	1 (0.1
Hypertension		1(0.1

* Numbers enclosed in parentheses in the first column indicate the number of women examined

### Birth outcome

More than half (53.1%; 333/627) delivered at home (Table 2). Most (98.1%; 607/627) were spontaneous vaginal deliveries, and Caesarean sections were performed on 1.3% (8/627). Information on the date of delivery for 6.7% (45/627) and place of delivery for 7.1% (48/627) was not obtained, as the mothers left the study area when their delivery date approached. Among newborns with available birth information on the date of delivery, 85.1% (536/630) delivered between the 37^th^ and 42^nd^ weeks, and 4.9% (31/630) were preterm. The mean birth weight was 3212.5 g (1292–5000 g), and 7.9% (48/610) were born with a low birth weight (Table 2).

### Intrauterine growth

Overall, 1,808 ultrasound measurements were taken. Most (90.8%; 613/675) had three ultrasound-derived weight measurements, and the remaining had one (n = 51) or two (n = 11) measurements only. The mean number of scans per foetus was 2.5, ranging from 1–4. Most 92% (621/675) of the fetal biometric measurements were taken at the second visit (25–27 weeks). Table 3 shows the distribution of ultrasound examinations and descriptive statistics of EFW by gestational age. It is worth noting that the coefficient of variation decreased as the number of ultrasound observations or measurements increased.

**Table 3:** Descriptive characteristics of estimated fetal weight and distribution of ultrasound examinations in relation to gestational age (Adami Tullu district, south-central Ethiopia, 2016–2017).

Gestational age (weeks)	Number of observations	Estimated fetal weight (g)			
		Mean ± SD	Minimum	Maximum	CV%
24	25	644 ± 36	575	718	5·6
25	36	769 ± 58	658	915	7·5
26	238	861±49	723	996	5·7
27	226	961+55	808	1130	5·7
28	80	1097 ± 67	952	1280	6·1
29	74	1263 ± 90	985	1471	7·1
30	208	1447 ±82	1236	1661	5·7
31	189	1589 ± 88	1362	1994	5·5
32	107	1765 ± 114	1465	2274	6·5
33	43	2024 ±175	1777	2902	8·6
34	61	2261 ± 116	2012	2528	5·1
35	133	2517 ± 131	2165	2808	5·2
36	249	2716 ± 131	2355	3058	4·8
37	100	2913 ± 156	2497	3395	5·3
38	27	3093 ± 260	2044	3555	8·4

CV, coefficient of variation (SD/mean), expressed as a percentage

**Fig 2:**
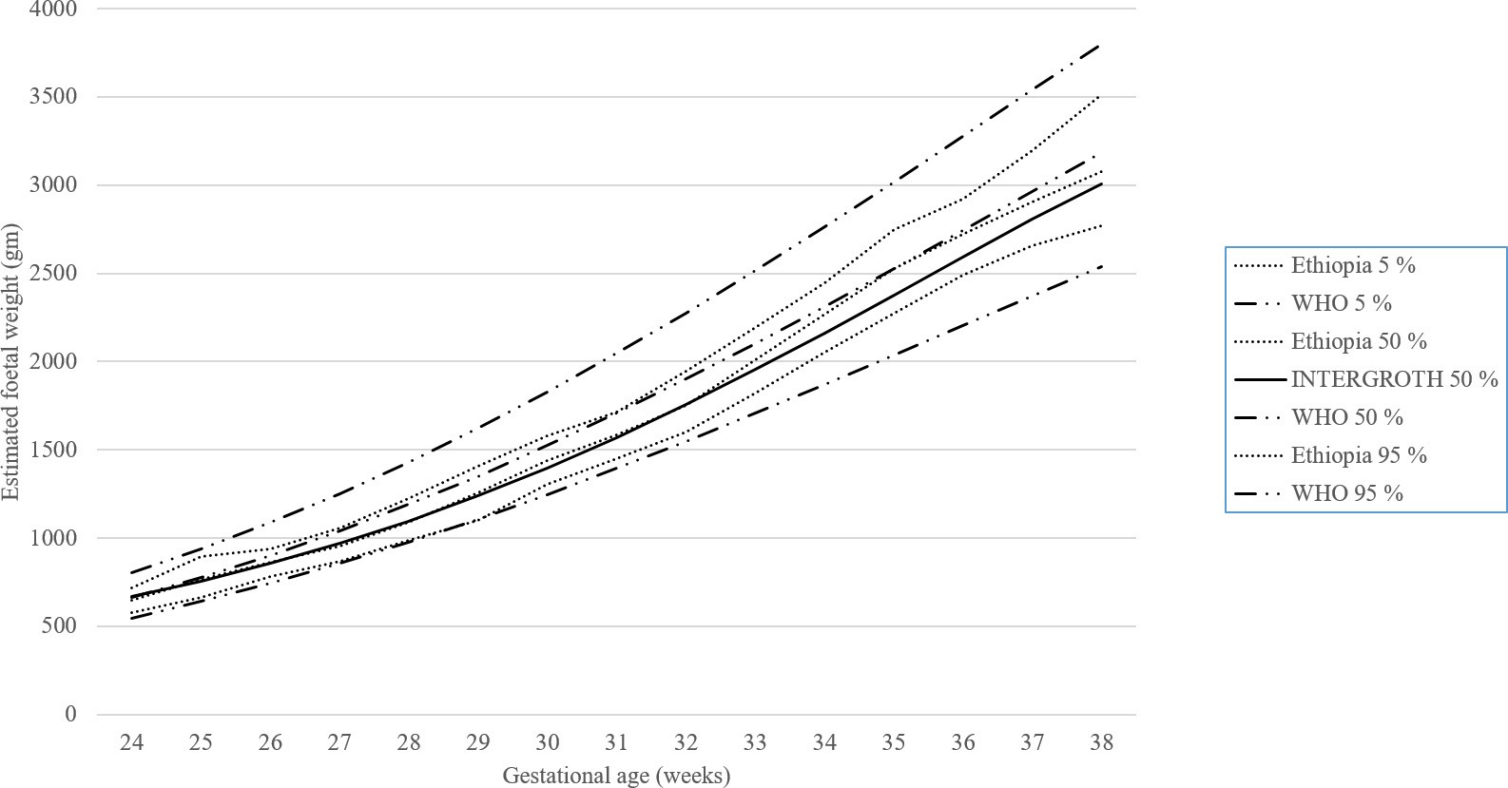
Distribution of estimated 5th, 50th, and 95th percentiles for fetal weight by study, WHO, and INTERGROWTH-21^st^ for gestational ages 24 to 38 weeks.

Fig 2 presents a chart for the EFWs of 1,808 ultrasound measurements compared to WHO and INTERGROWTH-21^st^ standards. This reference chart was plotted for the 5^th^, 50^th^, and 95^th^ percentiles for our study and WHO, and the 50^th^ percentile for INTERGROWTH-21^st^. We used the smoothed percentile to present the plot. As shown in the S1 Fig, the percentiles we calculated for EFW had similar distribution patterns as those for the WHO and INTERGROWTH-21st charts. The S2 Table shows comparisons of the 10^th^ and 90^th^ percentiles for the other biometric measurements and EFW.

Table 4 shows the corresponding reference values of our study percentiles of EFW, with respect to gestational age.

**Table 4:** Growth chart for estimated fetal weight percentiles for both sexes (Adami Tullu district, south-central Ethiopia, 2016–2017).

Gestational age (weeks)	Number of observations	Estimated fetal weight (g) by percentile						
		5^th^	10^th^	25^th^	50^th^	75^th^	90^th^	95^th^
**24**	25	580	599	614	645	663	710	717
**25**	36	663	697	735	765	802	848	893
**26**	238	781	795	828	865	897	921	936
**27**	226	871	892	924	957	994	1029	1058
**28**	80	985	1016	1053	1087	1141	1194	1226
**29**	74	1100	1148	1212	1257	1319	1371	1406
**30**	208	1304	1347	1395	1441	1502	1555	1582
**31**	189	1450	1479	1528	1585	1649	1695	1713
**32**	107	1600	1614	1696	1753	1838	1892	1946
**33**	43	1820	1843	1921	2011	2074	2165	2194
**34**	61	2051	2108	2181	2267	2341	2429	2449
**35**	133	2273	2350	2434	2527	2599	2689	2748
**36**	249	2492	2550	2627	2721	2811	2869	2923
**37**	100	2657	2730	2825	2905	3009	3081	3197
**38**	27	2367	2891	3033	3078	3232	3331	3515

The S3–S6 Tables show growth of the fetal outer-inner diameter of BPD, HC, AC, and FL percentiles with the corresponding gestational age. Overall, the EFW fell below the 10th centile (small for gestation) was 9.8% (177/1808) of the measurements in our study. The incidence of foetuses that were small for gestational age was the same for both sexes: 10% (95/951) for male and 10% (72/682) for female newborns. The S7 and S8 Tables show the corresponding reference value of percentiles of EFW for both sexes.

### Comparison with the INTERGROWTH-21st International fetal growth standard

**Fig 3:**
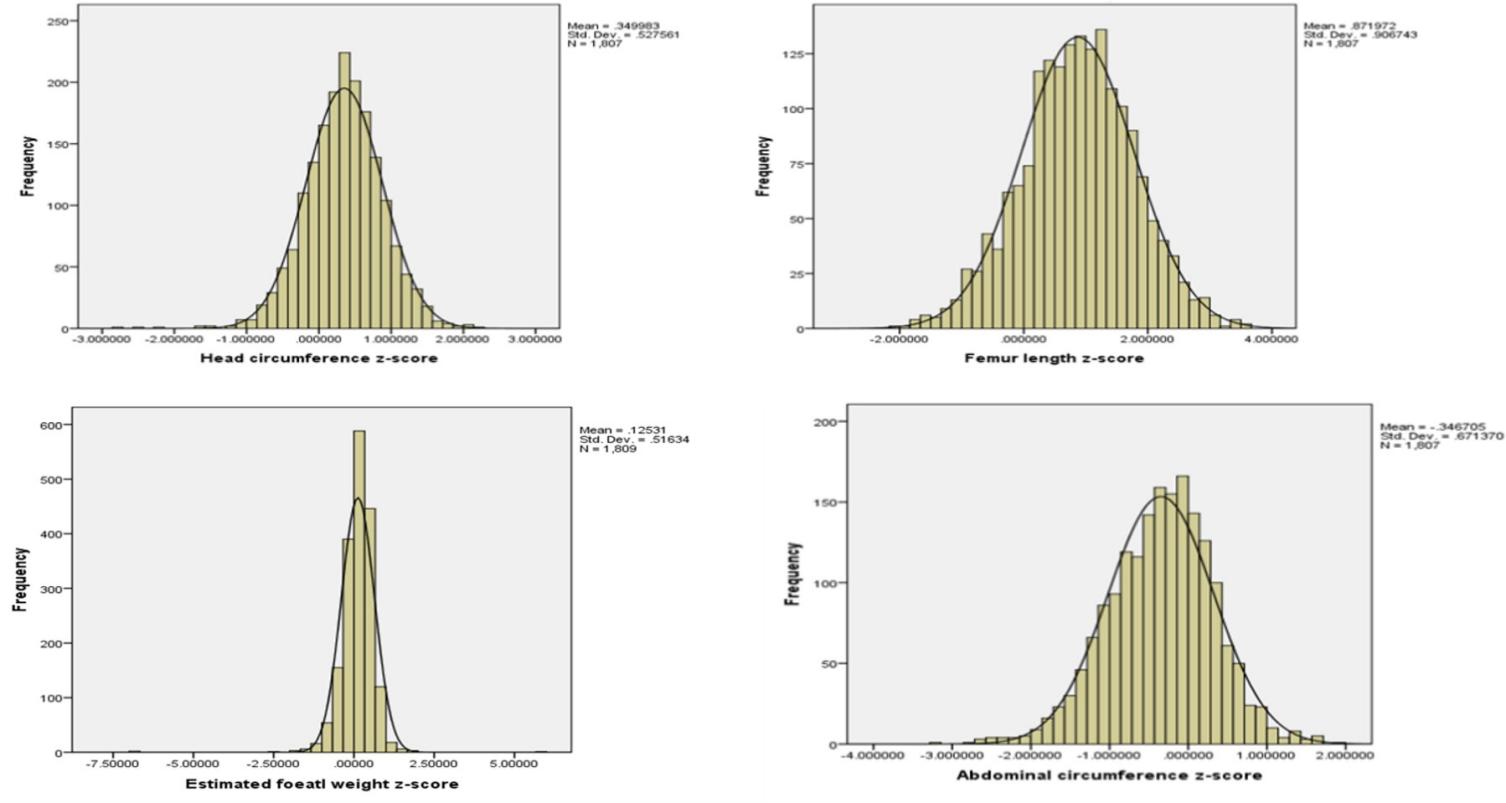
Z-score of biometric measurements and estimated fetal weight compared with the INTERGROWTH-21^st^ fetal growth standard, 2016–2017.

The z-score of the estimated fetal weight ranged from -2.36 to 1.89, with a mean of 0.13 and standard deviation of 0·44. Fig 3 and the S2 Fig show biometric measurements and EFW standardized z-scores that were calculated and compared with the INTERGROWTH-21^st^ fetal growth standard. Distribution of our biometric measurements for HC, FL, AC, and EFW were similar to those for the INTERGROWTH-21^st^ reference, and most measurements fell between -2 and +2 of the reference z-score. However, the z-score distribution of BPD was between -3 and +1 of the reference z-score.

## Discussion

We presented a fetal weight chart based on EFW using serial ultrasound measurements to elucidate fetal growth patterns in a drought-affected area of south-central Ethiopia. We generated a fetal growth chart using repeated growth assessments on the same foetus from a representative community sample of 675 singleton pregnancies without congenital malformations. Our main conclusion is that IUG patterns in the rural part of the Rift Valley are mostly within the same distributions as those from the WHO and INTERGROWTH-21^st^ multicentre fetal growth reference studies [9, 10, 31].

These findings indicate that, despite the presence of maternal under-nutrition in more than 40% of the women at the start of the study, the EFWs remained within normal ranges. A possible explanation for this could be that, although the area was affected by drought [32] and there was a food shortage resulting in under-nutrition, pregnant women in these areas received supplementary food. Indeed, pregnant mothers with MUACs < 23 cm do receive preferential and regular food supplements [22].

We also found that 9.8% of foetuses were small for gestational age, with a z-score for EFW below the 10^th^ percentile. This finding is suggestive of IUG restriction [13]. A study in Tanzania found similar results [33]. Compared to a previous study done in south western Ethiopia [18], we found a lower proportion of low birth weights (7.9%). This difference could be ascribed to the time that the birth weight was taken. In our study, 88.2% of birth weights were recorded within 24 hours of delivery.

Unlike previous reference studies done on presumably healthy and well-nourished pregnant women, our study was based on longitudinal ultrasound examinations in a population affected by drought. The availability of nutrients for the foetus depends on maternal nutrition. In drought-affected areas, low maternal food intake results in a reduced nutrient stream from the mother to the foetus, with consequent IUG restriction [13]. The presence of maternal undernutrition and consequent fetal growth restriction also might influence malnutrition rates among children younger than 5 years old, as previously reported in our study area by Taye et al. [34].

The z-scores of HC, FL, and AC in this study were similar to the standards, when fitted to INTERGROWTH-21^st^ application software [10, 35]. However, the z-score distribution of BPD was between -3 and +1 of the reference z-score. This difference is probably related to the difference in the method of BPD measurement in our study. Specifically, we measured BPD from outer to inner diameter of the parietal bone, whereas INTERGROWTH-21^st^ measures from outer to outer diameter. The difference in measurement between our study and INTERGROWTH-21^st^ is the thickness of the parietal bone, which is estimated to be less than 3 mm. This 3-mm difference is expected to shift the z-score to the right by at least one SD, as the SD in the INTERGROWTH-21^st^ study is approximately 1.74 mm. When accounting for the thickness of the parietal bone, the difference in BPD between our study and INTERGROWTH-21^st^ may not be significant [36].

Our study also differed from the INTERGROWTH-21^st^ study in determining EFW. Most studies, including WHO, use the Hadlock formula. For EFW, Hadlock 4 uses BPD (outer to inner diameter of the parietal bone), HC, AC, and FL. INTERGROWTH-21^st^ uses HC and AC only. In their study, Lufee Wong et al. concluded that EFW using Hadlock 3, Hadlock 4, and INTERGROWTH-21^st^ produces similar results, except for slight differences at gestations less than 27–28 weeks [37]. Previous studies have validated the use of Hadlock 3 and 4 formulae. The INTERGROWTH-21^st^ regression formula has yet to be prospectively validated against birth weights outside of the INTERGROWTH-21^st^ study [37].

Our study has many strengths. The study instruments were standardized and validated, and the women were representative of the community in this part of the Ethiopian Rift Valley. This population sample avoided possible selection bias, which can occur when data are collected at health facilities. The women were more likely to have an indication for follow-up or supervision. Moreover, longitudinal data produced reference intervals for both fetal size and growth, unlike cross-sectional studies that provide information on fetal size only. Migration of the study participants out of the study area as an effect of drought constitutes a possible limitation that could affect the results of this study. However, the proportion of those who migrated out of the study area was small (3.2%). Also, as our study evaluated EFWs at up to 36 weeks of gestation, the results might not determine whether growth faltering occurs in later gestational ages.

In conclusion, we measured IUG patterns using serial ultrasound measurements in a drought-affected rural community of Ethiopia and compared them with the INTERGROWTH-21^st^ and the WHO multicentre fetal growth reference standards. The fetal growth patterns, as measured by common ultrasound biometric measurements, were similar to the IUG patterns of the reference standards. The findings of our study also detected preterm births (4.9%) and low birth weights (7.9%) among participants. Subsequent studies on risk factors or underlying causes of preterm birth and low birth weight must be conducted to guide appropriate interventions.

## Supporting information

S1 Fig Growth chart of the 5^th^, 50^th^, and 95^th^ percentiles for our study and WHO reference standards and 50^th^ percentile for INTERGROWTH-21^st^ reference standards. (TIF)

S2 Fig Z-score of biparietal diameter compared with the INTERGROWTH-21^st^ fetal growth standard 2016–2017. (TIF)

S1 Table Comparison of baseline characteristics of those lost to follow up with those included in the final analysis. (DOCX)

S2 Table Growth chart of fetal outer-inner biparietal diameter at gestational ages 24–38 weeks (Adami Tullu district, south-central Ethiopia, 2016–2017). (DOCX)

S3 Table Growth chart of fetal head circumference (Adami Tullu district, south-central Ethiopia, 2016–2017). (DOCX)

S4 Table Growth chart of fetal abdominal circumference at gestational ages 24–38 weeks (Adami Tullu district, south-central Ethiopia, 2016–2017). (DOCX)

S5 Table Growth chart of fetal femur length at gestational ages 24–38 weeks (Adami Tullu district, south-central Ethiopia, 2016–2017). (DOCX)

S6 Table Growth chart of the 10^th^ and 90^th^ percentiles for our study and WHO reference standards. (DOCX)

S7 Table Estimated fetal weight for male newborns (Adami Tullu district, south-central Ethiopia, 2016–2017). (DOCX)

S8 Table Estimated fetal weight for female newborns (Adami Tullu district, south-central Ethiopia, 2016–2017). (DOCX)
