# Supplementary figures and images for: Intrauterine growth patterns in rural Ethiopia compared with WHO and INTERGROWTH-21st growth standards: A community-based longitudinal study
Source: PLoS One. 2019 Dec 31;14(12):e0226881. doi: 10.1371/journal.pone.0226881 (PMC6938373; doi:10.1371/journal.pone.0226881)

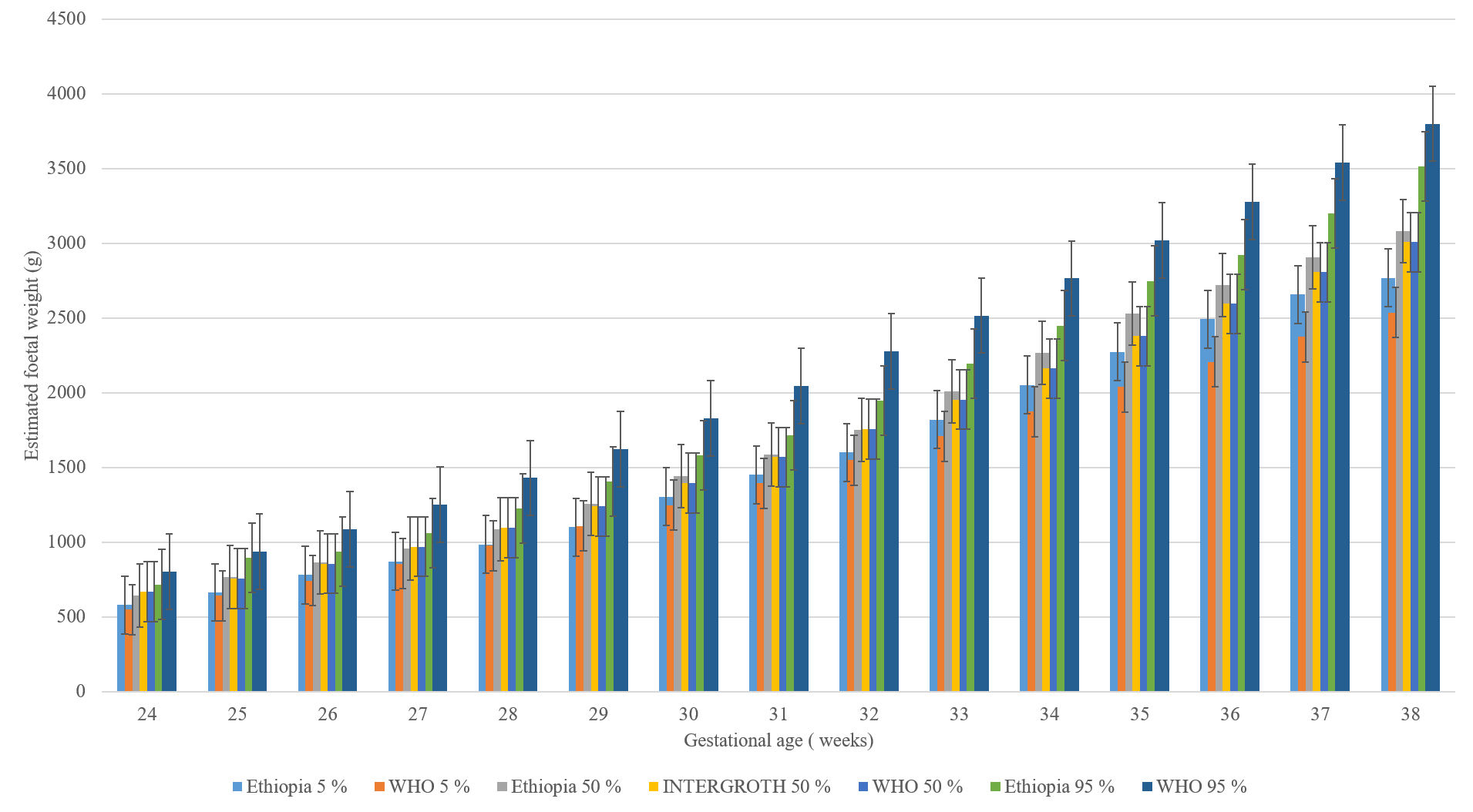

Supplement: S1 Fig — (TIF) [file pone.0226881.s001.tif]

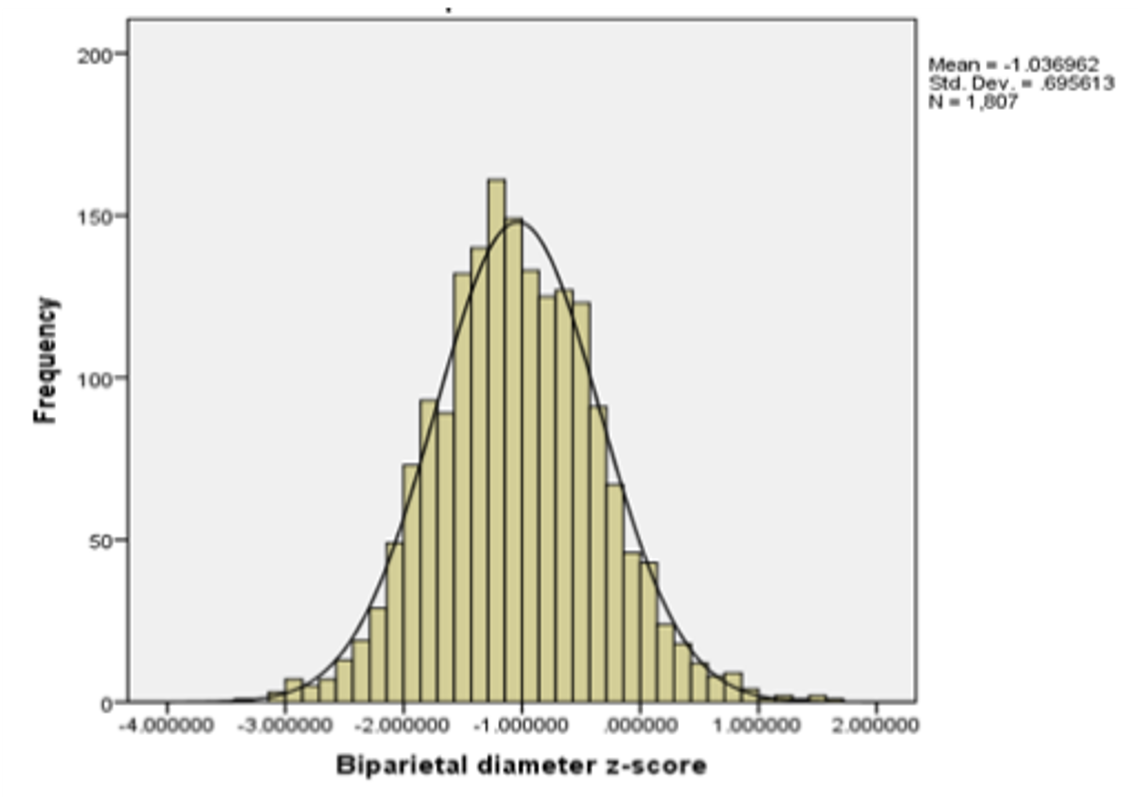

Supplement: S2 Fig — (TIF) [file pone.0226881.s002.tif]
