# Supplementary material for: Intrauterine growth patterns in rural Ethiopia compared with WHO and INTERGROWTH-21st growth standards: A community-based longitudinal study
Source: PLoS One. 2019 Dec 31;14(12):e0226881. doi: 10.1371/journal.pone.0226881 (PMC6938373; doi:10.1371/journal.pone.0226881)
Supplement: S1 Table — (DOCX) [file pone.0226881.s003.docx]

| **Variable** | **Loss to follow up** | | **P-value** |
| --- | --- | --- | --- |
|  | Yes (23, 3.3%) | No (666, 96.7%) |  |
| **Mean age (SD)** | 23.4 (5.3) | 24.9 (5.4) | P = .16  [t (687) = -1.4, two-tailed] |
| **Mean height** | 156.5 (5.9) | 157.3 (6.5) | P = .57  [t (687) = -.57, two-tailed] |
| **Occupation** |  | | |
| Others (%) | 5(3) | 159 (97) | P=0.90 (COR 1.07 (0.39- 2.94) |
| House wife | 17(3.2) | 507(96.8) | 1 |
| **Wealth status** |  | | |
| Poor (%) | 8 (3.6) | 216 (96.4) | 1 |
| Middle (%) | 8 (3.4) | 226 (96.6) | P=0.93 (COR 1.05 (0.39- 2.84) |
| Rich (%) | 5 (2.2) | 221 (97.8) | P=0.39 (COR 1.64 (0.53- 5.08) |
