# Supplementary material for: Intrauterine growth patterns in rural Ethiopia compared with WHO and INTERGROWTH-21st growth standards: A community-based longitudinal study
Source: PLoS One. 2019 Dec 31;14(12):e0226881. doi: 10.1371/journal.pone.0226881 (PMC6938373; doi:10.1371/journal.pone.0226881)
Supplement: S2 Table — (DOCX) [file pone.0226881.s004.docx]

| **Reference Chart** | **Measurement** | **Gestational Week** | | | |
| --- | --- | --- | --- | --- | --- |
|  |  | **24** | **28** | **32** | **36** |
| **10th percentile** | **Biparietal diameter mm)** |  |  |  |  |
|  | Ethiopia | 57 | 68 | 78 | 86 |
|  | WHO | 56 | 67 | 76 | 84 |
|  | **Head circumference (mm)** |  |  |  |  |
|  | Ethiopia | 217 | 253 | 289 | 320 |
|  | WHO | 211 | 251 | 282 | 306 |
|  | **Abdominal circumference (mm)** |  |  |  |  |
|  | Ethiopia | 177 | 218 | 257 | 299 |
|  | WHO | 184 | 225 | 260 | 294 |
|  | **Femural length (mm)** |  |  |  |  |
|  | Ethiopia | 40 | 49 | 58 | 68 |
|  | WHO | 41 | 49 | 57 | 65 |
|  | **Estimated foetal weight (g)** |  |  |  |  |
|  | Ethiopia | 599 | 1016 | 1614 | 2550 |
|  | WHO | 576 | 1026 | 1635 | 2352 |
| **90th percentile** | **Biparietal diameter mm)** |  |  |  |  |
|  | Ethiopia | 63 | 72 | 83 | 92 |
|  | WHO | 64 | 76 | 86 | 93 |
|  | **Head circumferance (mm)** |  |  |  |  |
|  | Ethiopia | 233 | 272 | 304 | 330 |
|  | WHO | 233 | 277 | 311 | 336 |
|  | **Abdominal circumference (mm)** |  |  |  |  |
|  | Ethiopia | 197 | 218 | 257 | 299 |
|  | WHO | 210 | 256 | 298 | 340 |
|  | **Femural length (mm)** |  |  |  |  |
|  | Ethiopia | 46 | 55 | 64 | 73 |
|  | WHO | 46 | 55 | 64 | 72 |
|  | **Estimated foetal weight (g)** |  |  |  |  |
|  | Ethiopia | 710 | 1194 | 1892 | 2869 |
|  | WHO | 765 | 1368 | 2187 | 3153 |
