# Supplementary material for: Intrauterine growth patterns in rural Ethiopia compared with WHO and INTERGROWTH-21st growth standards: A community-based longitudinal study
Source: PLoS One. 2019 Dec 31;14(12):e0226881. doi: 10.1371/journal.pone.0226881 (PMC6938373; doi:10.1371/journal.pone.0226881)
Supplement: S3 Table — (DOCX) [file pone.0226881.s005.docx]

| **Gestational age (weeks)** |  |  | **Biparietal diameter (mm) by percentile** | | | | | | |
| --- | --- | --- | --- | --- | --- | --- | --- | --- | --- |
|  | **Number of observations** | Mean + SD | **5^th^** | **10^th^** | **25^th^** | **50^th^** | **75^th^** | **90^th^** | **95^th^** |
| 24 | 25 | 59.5+ 1.7 | 57 | 57 | 58 | 59 | 60 | 63 | 63 |
| 25 | 36 | 61.03+2.3 | 56 | 58 | 60 | 61 | 63 | 64 | 65 |
| 26 | 238 | 64.6+ 1.7 | 61 | 62 | 64 | 65 | 66 | 67 | 67 |
| 27 | 226 | 67.1+1.9 | 64 | 64 | 66 | 67 | 69 | 69 | 70 |
| 28 | 80 | 69.9+ 1.9 | 66 | 68 | 68 | 70 | 71 | 72 | 73 |
| 29 | 74 | 72.2 + 2.2 | 69 | 70 | 71 | 72 | 74 | 75 | 75 |
| 30 | 208 | 75.0 + 1.9 | 72 | 73 | 74 | 75 | 76 | 77 | 78 |
| 31 | 189 | 77.6 + 2.2 | 74 | 75 | 76 | 78 | 79 | 81 | 81 |
| 32 | 107 | 80.2 + 2.1 | 76 | 78 | 79 | 80 | 82 | 83 | 84 |
| 33 | 43 | 83.3 + 2.5 | 79 | 80 | 82 | 83 | 85 | 86 | 87 |
| 34 | 61 | 84.0 + 2.2 | 81 | 81 | 83 | 84 | 86 | 88 | 88 |
| 35 | 133 | 86.2 + 2.4 | 82 | 83 | 84 | 86 | 88 | 89 | 90 |
| 36 | 249 | 89.0 + 2.2 | 86 | 86 | 87 | 89 | 90 | 92 | 92 |
| 37 | 100 | 91.1 + 2.7 | 86 | 88 | 90 | 91 | 93 | 94 | 95 |
| 38 | 27 | 94.4 + 2.8 | 87 | 91 | 93 | 95 | 96 | 98 | 99 |
|  |  |  |  |  |  |  |  |  |  |
