# Supplementary material for: Intrauterine growth patterns in rural Ethiopia compared with WHO and INTERGROWTH-21st growth standards: A community-based longitudinal study
Source: PLoS One. 2019 Dec 31;14(12):e0226881. doi: 10.1371/journal.pone.0226881 (PMC6938373; doi:10.1371/journal.pone.0226881)
Supplement: S4 Table — (DOCX) [file pone.0226881.s006.docx]

| **Gestational age (weeks)** | **Number of observations** | **Mean + SD** | **Head circumference (mm) by percentile** | | | | | | |
| --- | --- | --- | --- | --- | --- | --- | --- | --- | --- |
|  |  |  | **5^th^** | **10^th^** | **25^th^** | **50^th^** | **75^th^** | **90^th^** | **95^th^** |
| 24 | 25 | 223 +5.4 | 215 | 217 | 219 | 223 | 228 | 233 | 244 |
| 25 | 36 | 235 + 6.9 | 224 | 225 | 229 | 235 | 240 | 242 | 247 |
| 26 | 238 | 244.1+ 4.3 | 237 | 239 | 241 | 244 | 247 | 250 | 251 |
| 27 | 226 | 252 +4.7 | 244 | 246 | 249 | 252 | 255 | 258 | 260 |
| 28 | 80 | 262.1+ 6.5 | 252 | 253 | 258 | 262 | 265 | 272 | 274 |
| 29 | 74 | 272..3+ 8.0 | 260 | 263 | 268 | 273 | 277 | 281 | 284 |
| 30 | 208 | 281 + 5.0 | 273 | 275 | 277 | 281 | 284 | 287 | 289 |
| 31 | 189 | 288.8 + 5.8 | 280 | 281 | 285 | 288 | 293 | 296 | 300 |
| 32 | 107 | 296.6 + 6.2 | 285 | 289 | 292 | 297 | 301 | 304 | 308 |
| 33 | 43 | 305 + 7.0 | 292 | 297 | 301 | 303 | 309 | 313 | 315 |
| 34 | 61 | 313.9 + 5.6 | 305 | 307 | 310 | 313 | 318 | 322 | 324 |
| 35 | 133 | 319.1+ 4.7 | 312 | 313 | 316 | 319 | 322 | 324 | 326 |
| 36 | 249 | 325.3+ 4.3 | 318 | 320 | 323 | 325 | 328 | 330 | 332 |
| 37 | 100 | 329.7+ 6.1 | 320 | 324 | 326 | 330 | 333 | 338 | 339 |
| 38 | 27 | 334.9 + 6.9 | 317 | 329 | 331 | 335 | 338 | 344 | 345 |
