# Supplementary material for: Intrauterine growth patterns in rural Ethiopia compared with WHO and INTERGROWTH-21st growth standards: A community-based longitudinal study
Source: PLoS One. 2019 Dec 31;14(12):e0226881. doi: 10.1371/journal.pone.0226881 (PMC6938373; doi:10.1371/journal.pone.0226881)
Supplement: S5 Table — (DOCX) [file pone.0226881.s007.docx]

|  | **Number of observations** | **Mean + SD** | **Abdominal circumference (mm) by percentile** | | | | | | |
| --- | --- | --- | --- | --- | --- | --- | --- | --- | --- |
| **Gestational age (weeks)** |  |  | **5^th^** | **10^th^** | **25^th^** | **50^th^** | **75^th^** | **90^th^** | **95^th^** |
| 24 | 25 | 187.8 + 7.8 | 170 | 177 | 184 | 189 | 195 | 197 | 199 |
| 25 | 36 | 200.3 + 9.2 | 185 | 191 | 194 | 199 | 207 | 216 | 218 |
| 26 | 238 | 208.6 +7.9 | 196 | 199 | 203 | 209 | 215 | 218 | 222 |
| 27 | 226 | 217+ 7.6 | 205 | 208 | 212 | 216 | 222 | 227 | 231 |
| 28 | 80 | 227.4 + 9.3 | 210 | 218 | 222 | 227 | 232 | 237 | 244 |
| 29 | 74 | 238.5 + 10.6 | 215 | 224 | 231 | 238 | 245 | 253 | 256 |
| 30 | 208 | 249.4 + 8.6 | 235 | 238 | 243 | 249 | 256 | 260 | 264 |
| 31 | 189 | 257.6 + 8.5 | 245 | 246 | 252 | 258 | 263 | 269 | 272 |
| 32 | 107 | 268.1 + 10.4 | 252 | 257 | 261 | 268 | 276 | 280 | 285 |
| 33 | 43 | 280.1 + 11.0 | 263 | 269 | 273 | 279 | 286 | 290 | 294 |
| 34 | 61 | 290.3 + 9.8 | 276 | 279 | 284 | 289 | 296 | 307 | 311 |
| 35 | 133 | 302.7 + 9.4 | 285 | 289 | 297 | 303 | 309 | 314 | 317 |
| 36 | 249 | 311.1 + 8.8 | 295 | 299 | 306 | 312 | 317 | 322 | 325 |
| 37 | 100 | 317.9 + 10.7 | 298 | 305 | 312 | 319 | 323 | 330 | 334 |
| 38 | 27 | 325.2 + 13.9 | 285 | 314 | 322 | 326 | 334 | 338 | 342 |
