# Supplementary material for: Intrauterine growth patterns in rural Ethiopia compared with WHO and INTERGROWTH-21st growth standards: A community-based longitudinal study
Source: PLoS One. 2019 Dec 31;14(12):e0226881. doi: 10.1371/journal.pone.0226881 (PMC6938373; doi:10.1371/journal.pone.0226881)
Supplement: S6 Table — (DOCX) [file pone.0226881.s008.docx]

| **Gestational age (weeks)** | **Number of observations** | **Mean + SD** | **Femur length (mm) by percentile** | | | | | | |
| --- | --- | --- | --- | --- | --- | --- | --- | --- | --- |
|  |  |  | **5^th^** | **10^th^** | **25^th^** | **50^th^** | **75^th^** | **90^th^** | **95^th^** |
| 24 | 25 | 43.3 **+** 2.2 | 39 | 40 | 42 | 43 | 45 | 46 | 47 |
| 25 | 36 | 46.6 **+** 2.2 | 43 | 44 | 46 | 47 | 48 | 49 | 50 |
| 26 | 238 | 48.1 + 1.8 | 45 | 46 | 47 | 48 | 49 | 50 | 51 |
| 27 | 226 | 50 + 1.7 | 47 | 48 | 49 | 50 | 51 | 52 | 52 |
| 28 | 80 | 52.1 + 2.1 | 48 | 49 | 51 | 52 | 53 | 55 | 56 |
| 29 | 74 | 54.8 + 2.2 | 51 | 52 | 53 | 55 | 56 | 57 | 58 |
| 30 | 208 | 57.4 + 1.8 | 54 | 55 | 56 | 57 | 59 | 60 | 61 |
| 31 | 189 | 59 + 2.0 | 55 | 57 | 58 | 59 | 60 | 61 | 62 |
| 32 | 107 | 60.7 + 2.2 | 57 | 58 | 59 | 61 | 62 | 64 | 65 |
| 33 | 43 | 63.8 + 2.7 | 60 | 61 | 62 | 63 | 65 | 67 | 68 |
| 34 | 61 | 66.3 + 2.6 | 62 | 63 | 64 | 66 | 68 | 69 | 71 |
| 35 | 133 | 68.5 + 2.3 | 64 | 65 | 67 | 69 | 70 | 71 | 72 |
| 36 | 249 | 70.2 + 2.0 | 67 | 68 | 69 | 70 | 71 | 73 | 74 |
| 37 | 100 | 72 + 2.1 | 69 | 70 | 70 | 72 | 73 | 75 | 76 |
| 38 | 27 | 72.9 + 2.8 | 68 | 69 | 71 | 73 | 75 | 77 | 78 |
