# Supplementary material for: Intrauterine growth patterns in rural Ethiopia compared with WHO and INTERGROWTH-21st growth standards: A community-based longitudinal study
Source: PLoS One. 2019 Dec 31;14(12):e0226881. doi: 10.1371/journal.pone.0226881 (PMC6938373; doi:10.1371/journal.pone.0226881)
Supplement: S7 Table — (DOCX) [file pone.0226881.s009.docx]

| **Gestational**  **age (weeks)** | **Number of observations** | **Male estimated foetal weight (g) by percentile** | | | | | | |
| --- | --- | --- | --- | --- | --- | --- | --- | --- |
|  |  | 5^th^ | 10^th^ | 25^th^ | 50^th^ | 75^th^ | 90^th^ | 95^th^ |
| 24 | 25 | 575 | 580 | 610 | 633 | 666 | 693 | 732 |
| 25 | 36 | 664 | 677 | 728 | 753 | 809 | 877 | 926 |
| 26 | 238 | 776 | 792 | 818 | 857 | 904 | 914 | 947 |
| 27 | 226 | 860 | 884 | 917 | 958 | 1008 | 1025 | 1060 |
| 28 | 80 | 989 | 1025 | 1053 | 1085 | 1131 | 1191 | 1231 |
| 29 | 74 | 997 | 1119 | 1204 | 1243 | 1328 | 1332 | 1377 |
| 30 | 208 | 1309 | 1349 | 1393 | 1437 | 1509 | 1554 | 1570 |
| 31 | 189 | 1442 | 1478 | 1519 | 1578 | 1658 | 1696 | 1735 |
| 32 | 107 | 1601 | 1612 | 1686 | 1741 | 1818 | 1895 | 1934 |
| 33 | 43 | 1777 | 1809 | 1872 | 1944 | 2115 | 2097 | 2213 |
| 34 | 61 | 2027 | 2076 | 2152 | 2267 | 2327 | 2429 | 2435 |
| 35 | 133 | 2274 | 2319 | 2435 | 2515 | 2648 | 2658 | 2790 |
| 36 | 249 | 2502 | 2540 | 2605 | 2698 | 2817 | 2858 | 2887 |
| 37 | 100 | 2656 | 2745 | 2817 | 2915 | 3017 | 3084 | 3202 |
| 38 | 27 | 2680 | 2610 | 2975 | 3075 | 3283 | 3346 | 3532 |
