# Supplementary material for: Intrauterine growth patterns in rural Ethiopia compared with WHO and INTERGROWTH-21st growth standards: A community-based longitudinal study
Source: PLoS One. 2019 Dec 31;14(12):e0226881. doi: 10.1371/journal.pone.0226881 (PMC6938373; doi:10.1371/journal.pone.0226881)
Supplement: S8 Table — (DOCX) [file pone.0226881.s010.docx]

| **Gestational**  **age (weeks)** | **Number of observations** | **Female estimated foetal weight (g) by percentile** | | | | | | |
| --- | --- | --- | --- | --- | --- | --- | --- | --- |
|  |  | 5^th^ | 10^th^ | 25^th^ | 50^th^ | 75^th^ | 90^th^ | 95^th^ |
| 24 | 25 | 604 | 604 | 621 | 653 | 650 | 678 | 744 |
| 25 | 36 | 658 | 676 | 743 | 766 | 799 | 880 | 919 |
| 26 | 238 | 789 | 809 | 838 | 869 | 888 | 921 | 930 |
| 27 | 226 | 885 | 907 | 932 | 957 | 997 | 1044 | 1073 |
| 28 | 80 | 989 | 1011 | 1060 | 1104 | 1144 | 1194 | 1238 |
| 29 | 74 | 1125 | 1210 | 1250 | 1303 | 1313 | 1420 | 1466 |
| 30 | 208 | 1305 | 1333 | 1400 | 1455 | 1494 | 1564 | 1587 |
| 31 | 189 | 1458 | 1499 | 1552 | 1602 | 1645 | 1693 | 1704 |
| 32 | 107 | 1596 | 1609 | 1701 | 1753 | 1837 | 1876 | 1886 |
| 33 | 43 | 1859 | 1907 | 1967 | 2012 | 2059 | 2202197 | 2324 |
| 34 | 61 | 2139 | 2177 | 2186 | 2251 | 2371 | 2435 | 2453 |
| 35 | 133 | 2246 | 2329 | 2437 | 2531 | 2576 | 2691 | 2747 |
| 36 | 249 | 2470 | 2554 | 2647 | 2750 | 2782 | 2879 | 2926 |
| 37 | 100 | 2506 | 2728 | 2826 | 2876 | 3005 | 3104 | 3302 |
| 38 | 27 | 3016 | 3016 | 3038 | 3119 | 3226 | 3385 | 3630 |
